# Gestational Outcomes of Pregnant Women Who Have Had Invasive Prenatal Testing for the Prenatal Diagnosis of Duchenne Muscular Dystrophy

**DOI:** 10.1155/2018/9718316

**Published:** 2018-07-30

**Authors:** Mehmet Sinan Beksac, Atakan Tanacan, Duygu Aydin Hakli, Gokcen Orgul, Burcu Soyak, Burcu Balci Hayta, Pervin Dincer, Haluk Topaloğlu

**Affiliations:** ^1^Division of Perinatology, Department of Obstetrics and Gynecology, Hacettepe University Faculty of Medicine, Ankara, Turkey; ^2^Department of Biostatistics, Hacettepe University Faculty of Medicine, Ankara, Turkey; ^3^Department of Medical Biology, Hacettepe University Faculty of Medicine, Ankara, Turkey; ^4^Division of Neurology, Department of Pediatrics, Hacettepe University Faculty of Medicine, Ankara, Turkey

## Abstract

**Aim:**

To show the importance of prenatal diagnosis of Duchenne Muscular Dystrophy (DMD) and to demonstrate the effect of DMD gene mutations on gestational outcomes.

**Materials and Methods:**

We retrospectively evaluated 89 pregnancies in 81 individuals who were referred to Hacettepe University for prenatal diagnosis of DMD between January 2000 and December 2015. Prenatal diagnostic methods (chorionic villus sampling (CVS): 66, amniocentesis (AC): 23) were compared for test results, demographic features, and obstetric outcomes of pregnancies. The female fetuses were divided into two groups according to the DMD status (healthy or carrier) to understand the effect of DMD gene mutations on obstetric outcomes.

**Results:**

Eight prenatally diagnosed disease-positive fetuses were terminated. There was no statistically significant difference between the CVS and AC groups in terms of study variables. There were 46 male fetuses (51.6%) and 43 female fetuses (48.4%). Fifteen of the female fetuses were carriers (34.8%). Median birthweight values were statistically insignificantly lower in the carrier group.

**Conclusion:**

Pregnancies at risk for DMD should be prenatally tested to prevent the effect of disease on families and DMD carrier fetuses had obstetric outcomes similar to DMD negative female fetuses.

## 1. Introduction

Duchenne muscular dystrophy (DMD) is an X-linked recessive disease with an incidence of 1 in 3500 live male births [1, 2]. Mutations in the DMD gene lead to progressive and symmetrical wasting of proximal muscles beginning from the lower limbs, and the affected children are wheelchair bound by the age of approximately 12 years. Thereafter, most patients die owing to cardiomyopathy and/or respiratory complications in their adult life [1–3].

DMD gene is one of the largest human genes identified, which is composed of 79 exons, spanning 2.4 Mb of DNA in Xp21 [3]. The majority of DMD gene mutations are deletions (60-65%), followed by duplications (8-15%), and the remaining are composed of microdeletions/microinsertions, missense mutations, nonsense mutations, splicing mutations, and deep intronic mutations [3, 4]. Although mutation detection may be difficult due to the large size of the gene, multiplex ligation-dependent probe amplification (MLPA) technology has eased the diagnosis [3, 5, 6].

Since there is currently no effective treatment for the disease, antenatal genetic counseling and prenatal diagnosis are vital [7]. Chorionic villus sampling (CVS) and amniocentesis (AC) are commonly used invasive prenatal testing (IPTs) for the prenatal diagnosis of DMD. Preimplantation Genetic Diagnosis (PGD) may be another alternative [8]. Besides, cell-free fetal DNA (cffDNA) has been in practice during the recent years, but it is not a routine part of the prenatal screening owing to various limitations [9]. Prenatal diagnosis of DMD provides an option of pregnancy termination and an opportunity to deliver disease-positive infants at tertiary healthcare centers with a multidisciplinary approach. Additionally, short stature and delayed puberty were reported to be more frequent in patients with DMD [10, 11]. Boys with DMD have a slower growth velocity during the first years of life and they are within the lower percentiles at childhood and adolescence [10, 11]. Although the exact aetiology behind this growth retardation has not been revealed yet, deletions of the Short Stature Homeobox (SHOX) gene which is located near to Xp21, abnormal response of dystrophic muscle to endogenous growth hormone, and application of corticosteroid therapies may be the reasons for this situation [10, 11]. However, to the best of our knowledge, there is no study in the literature that compares the obstetric outcomes of healthy and carrier female fetuses in terms of DMD. Thus, we compared the obstetric outcomes of the healthy and carrier female fetuses.

In this study, we share our experience of prenatal diagnosis and management of DMD at Hacettepe University Hospital Perinatology Division.

## 2. Materials and Methods

We retrospectively evaluated 89 pregnancies in 81 individuals who were referred to the Division of Perinatal Medicine, Hacettepe University, Ankara, for the prenatal diagnosis of DMD between January 2000 and December 2015. Data consisted of patients' demographic features, obstetric history, IPTs used, and obstetric outcomes, which were obtained from the Hacettepe University Perinatal Medicine database. Pregnancies in women related to patients with other muscular dystrophies or congenital myopathies were excluded from the study.

Prenatal diagnosis was performed in couples with mothers being carriers of the disease or at least one child with DMD in their families. CVS was performed in pregnancies between the 11th and 14th gestational weeks, and AC was performed in pregnancies between the 16th and 20th gestational weeks, under ultrasound guidance. Chorion villus specimens were dissected under the microscope to remove maternal tissue and were transferred to the Department of Medical Biology for molecular genetic analysis. Fetal DNA was extracted from uncultured chorionic villus and amniotic fluid using the standard phenol-chloroform method. Fetal sex was determined by amelogenin gene amplification. Multiplex PCR was performed in male fetuses for the rapid detection of hot spot exonal deletions [12, 13]. In addition, DNA sequencing was used in male fetuses with known familial point mutation; linkage analysis was performed for male and female fetuses to track the mutant chromosome in family members. Additionally, multiplex ligation-dependent probe amplification (MLPA) was performed according to instructions from the manufacturer (MRC-Holland) for the detection of deletions and duplications in affected cases for the last two years.

Prenatal diagnostic methods (66 CVS, 23 AC) were compared for the test results, demographic features, and the obstetric outcomes of the pregnancies. Female fetuses were divided into two groups according to the DMD status (healthy or carrier) to understand the effect of DMD gene mutations on obstetric outcomes. The two groups were compared for the mode of delivery, percentage of diagnostic tests used, age, gravida, and parity of the mothers; gestational week at birth, birthweight, and 5th minute APGAR scores of the infants; and cesarean (CS) rates. The study protocol has been approved by the institutional ethics committee of Hacettepe University (GO16/690).

Statistical analysis was conducted using Statistical Package for the Social Sciences (SPSS.22®). The mean and standard deviation values along with the median, minimum, and maximum values were calculated and compared between the groups according to homogeneity of distribution among the variables, and the percentages were calculated where necessary.

## 3. Results

The mean age of the patients in 89 pregnancies was 30.1 ± 5.6 years, the mean gravida was 3.0 ± 1.4, the mean parity was 1.3 ± 1.0, and the mean gestational week at the time of IPT (CVS plus AC) was 13.2 ± 2.2 weeks.

There were eight prenatally diagnosed disease-positive fetuses in 89 pregnancies. All DMD cases were terminated after genetic counseling and ethical and legal permissions. The mean gestational week of pregnancy termination was 16 ± 2.6 weeks. CVS was performed in 66 patients (74.1%), and AC was performed in the remaining 23 patients (25.9%). In the CVS group, 51 fetuses were healthy (77.2%), nine fetuses were carriers (13.6%), and six fetuses were disease-positive (9.2%). In the AC group, 15 fetuses were healthy (65.2%), six fetuses were carriers (26.0%), and two fetuses were disease- positive (8.8%). There was no statistically significant difference between the CVS and AC groups for the median maternal age, gravida, parity, gestational week at birth, birthweight, and 5th minute APGAR scores. The median gestational weeks of the prenatal test for the CVS and AC groups were 12 (11 to 14) and 16 (16 to 21) weeks, respectively. We did not observe intervention related complications in both groups.

Table 1 shows the percentages, demographic features, and prenatal diagnostic test results of the pregnancies in CVS and AC groups, represented as median and minimum-maximum values.

Table 2 shows the obstetric outcomes of the pregnancies in CVS and AC groups, represented as median and minimum-maximum values. There were 46 male fetuses (51.6%) and 43 female fetuses (48.4%) in 89 pregnancies. Eight of the male fetuses were DMD positive (17.4%) and 38 were healthy (82.6%); 15 of the female fetuses were carriers (34.8%). Number of healthy fetuses is higher than expected in our study. This is most probably due to the relatively small number of individuals in the study group.  Table 3 shows the demographic features, prenatal diagnostic tests used, and the obstetric outcomes of the pregnancies in the healthy and carrier female fetus groups as percentage, median, minimum, and maximum values. Although the median birthweight was slightly lower in the carrier group, there was no statistically significant difference between the variables among the groups.

We also compared the obstetric outcomes of the female healthy and carrier fetuses with healthy male fetuses. The median birth week of healthy male fetuses was 39 weeks and the median birthweight of the healthy male infants was 3150 g. In addition, the median 5th minute APGAR scores was 10. CS rate was 68.4% (26/38). Although median birth week and birthweight of the healthy male infants were higher than the female carrier fetuses; there was no statistically significant difference between the groups (p=0.60).

## 4. Discussion

Prenatal diagnosis of DMD is crucial because this disease has the most severe clinical symptoms among X-linked recessive inherited muscular dystrophies, and no curative treatment is currently available [1]. The main targets of therapy are to prevent respiratory and cardiac complications and to maintain quality of life with supportive care. Glucocorticoid treatment is the most commonly applied therapy to reduce the symptoms [9]. There are also novel therapies like gene therapy, eteplirsen (an antisense oligonucleotide), ataluren (an investigational orally administered drug), creatinine, myostatin inactivation, cell therapy, and idebenone, but none of them have yet provided a definitive treatment of DMD [14–20]. Considering the poor prognosis for patients and high cost of disease management for families, a prenatal diagnosis seems to be the most reasonable approach at present.

CVS was the first choice for prenatal diagnosis at our institution (74.1%) as the results could be obtained during an early gestational week; thus, termination of the pregnancy could be performed earlier if the fetus was disease-positive. Due to the high rate of CVS, the mean gestational age at the time of IPTs was 13.2 ± 2.2 weeks. However, the mean gestational age at pregnancy termination was 16 ± 2.6 weeks. This time interval was due to the delay in decision taken by the families for the termination of pregnancy. Since it was difficult for the families to make an exact decision, we supported them through the whole process, and appropriate consultations were organized in a multidisciplinary approach. All of the eight disease-positive pregnancies were terminated after obtaining ethical and legal permissions. This high rate might be the result of informing the patients accurately and objectively about the diagnosis and treatment options of DMD and its devastating impact on the economic, social, and emotional lives of families.

Although invasive prenatal procedures are associated with an estimated 0.5-1% risk of pregnancy loss [14, 15], there was no procedure-related loss in our study. Carrier frequency of DMD was 34.8% in our study. Although most of the carriers are asymptomatic, there is clinically apparent muscle [21] weakness in approximately 2.5 to 20 percent of carriers, and an elevated serum creatine kinase concentration in up to 70% of them [16]. However, an early onset progressive muscular dystrophy could be seen in carriers with 45 X, 46 XY, and mosaic Turner karyotypes, apparently balanced X/autosome translocations with breakpoints in Xp21 within the dystrophin gene, and preferential inactivation of the normal X and nonrandom (skewed) X-chromosome inactivation that leads to diminished expression of the normal dystrophin allele [17, 22, 23]. Cardiology counseling and close follow-up for cardiomyopathy are mandatory for these patients, as dilated cardiomyopathy is reported in 8 percent of carriers [7, 18, 23].

Gestational age at birth, birthweight, and the 5th minute APGAR scores were calculated and compared for the prenatal diagnostic invasive procedure groups, and all the variables were found to be within normal ranges. Although IPTs may affect birthweights of the infants [24], the type of the IPTs had no effect on pregnancy outcomes in our study.

The male fetus DMD ratio (17.4%) was higher than expected according to the literature [2], but this might be an effect of the small number of high risk DMD pregnancies that were evaluated in our study.

To our knowledge, there is no current study in the literature that compares the obstetric outcomes of healthy and carrier female fetuses in terms of DMD. Carrier and healthy female fetuses were evaluated, and there was no statistically significant difference between the variables among the groups. Although the median gestational week at birth was earlier and the median birthweight was lower in the carrier group, there was no statistically significant difference. However, multicenter randomized controlled trials with large patient populations are necessary for further assessment.

In conclusion, IPTs performed at experienced centers are safe, and pregnancies at risk for DMD should be prenatally tested to prevent the effect of disease on the families and the healthcare system. DMD carrier fetuses had obstetric outcomes similar to DMD negative female fetuses; however, close follow-up for the management of complications, like muscle weakness and cardiomyopathy, should be planned for these patients. Currently, invasive prenatal diagnostic tests are mostly used, but with the advances in cffDNA technology, noninvasive tests could become the standard test within a short period of time [9, 19, 20].

## Figures and Tables

**Table 1:** Percentages, demographic features, and prenatal diagnostic test results of the pregnancies in chorionic villus sampling (CVS) and amniocentesis (AC) groups, represented as median and minimum-maximum values.

Prenatal Diagnostic Procedure	CVS (n=66)	AC (n=23)	P value
Percentage	74.1%	25.9%	

Age (years) of mother	31 (19 to 43)	28 (18 to 41)	0.256

Gravida	3 (1 to 7)	1 (1 to 4)	0.890

Parity	1 (0 to 4)	1 (0 to 4)	0.822

Gestational week of the prenatal test	12 (11 to 14)	16 (16 to 21)	< 0.001

Disease-positive fetuses	6 (9.2%)	2 (8.8%)	

CVS: chorion villus sampling and AC: amniocentesis.

**Table 2:** Obstetric outcomes of the pregnancies in chorionic villus sampling (CVS) and amniocentesis (AC) groups, represented as median and minimum-maximum values.

Prenatal Diagnostic Procedure	CVS (n=66)	AC (n=23)	P value
Gestational week at birth	39 (37 to 40)	39 (37 to 41)	0.916

Birthweight (grams)	3100 (2600 to 3800)	3200 (2650 to 3900)	0.742

5^th^minute APGAR	10 (7 to 10)	10 (7 to 10)	0.916

CS Rate	38/60 (63.3%)	13/21 (61.9%)	

**Table 3:** Percentages, demographic features, prenatal diagnostic tests, and obstetric outcomes of the pregnancies in healthy and carrier female fetus groups, represented as median and minimum-maximum values.

DMD status	Healthy (n=28)	Carrier (n=15)	P value
Percentage	65.2%	34.8%	

CVS	24 (85.8%)	9 (60%)	

Amniocentesis	4 (14.2%)	6 (40%)	

Age (years) of mother	30 (20 to 43)	31 (19 to 41)	1.0

Gravida	3 (1 to 7)	3 (1 to 7)	0.574

Parity	1 (0 to 3)	1 (0 to 4)	0.285

Gestational week at birth	39 (37 to 40)	38 (37 to 41)	0.596

Birthweight (grams)	3250 (2600 to 3800)	2900 (2500 to 3100)	0.596

5^th^ minute APGAR	10 (7 to 10)	8 (7 to 10)	0.825

CS rate	14/28 (50%)	11/15 (73.3%)	

CVS: Chorion villus sampling, AC: Amniocentesis, CS: Cesarean.
